# Supplementary figures and images for: Bi-fated tendon-to-bone attachment cells are regulated by shared enhancers and KLF transcription factors
Source: eLife. 2021 Jan 15;10:e55361. doi: 10.7554/eLife.55361 (PMC7810463; doi:10.7554/eLife.55361)

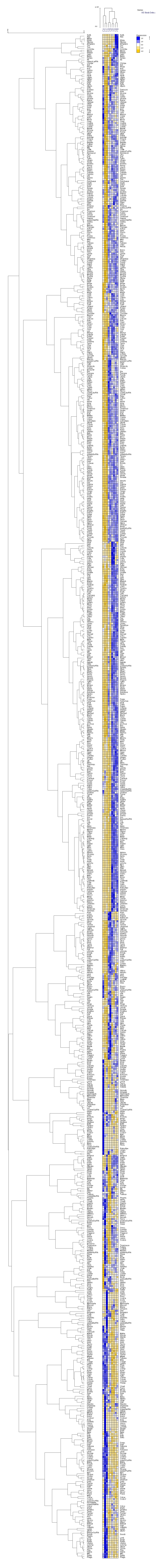

Supplement: Supplementary file 1. — Ordered genes and samples were combined by complete-linkage clustering using the similarity measurement of Pearson correlation. A, remote tenocytes; B, adjacent tenocytes; C, remote chondrocytes; D, adjacent chondrocytes; E, attachment cells. [file elife-55361-supp1.jpg]
