# Supplementary material for: Bi-fated tendon-to-bone attachment cells are regulated by shared enhancers and KLF transcription factors
Source: eLife. 2021 Jan 15;10:e55361. doi: 10.7554/eLife.55361 (PMC7810463; doi:10.7554/eLife.55361)
Supplement: Transparent reporting form [file elife-55361-transrepform.docx]

***eLife’s* transparent reporting form**

We encourage authors to provide detailed information *within their submission* to facilitate the interpretation and replication of experiments. Authors can upload supporting documentation to indicate the use of appropriate reporting guidelines for health-related research (see [EQUATOR Network](http://www.equator-network.org/%20)), life science research (see the [BioSharing Information Resource](https://biosharing.org/)), or the [ARRIVE guidelines](http://www.plosbiology.org/article/info:doi/10.1371/journal.pbio.1000412) for reporting work involving animal research. Where applicable, authors should refer to any relevant reporting standards documents in this form.

If you have any questions, please consult our Journal Policies and/or contact us: [editorial@elifesciences.org](mailto:editorial@elifesciences.org).

**Sample-size estimation**

- You should state whether an appropriate sample size was computed when the study was being designed
- You should state the statistical method of sample size computation and any required assumptions
- If no explicit power analysis was used, you should describe how you decided what sample (replicate) size (number) to use

Please outline where this information can be found within the submission (e.g., sections or figure legends), or explain why this information doesn’t apply to your submission:

Sample-size estimates do not apply to this study. This study was done by epigenetic profiling and transcriptome analysis of mouse tissues, and compared to existing mouse epigenetic and genetic data.

**Replicates**

- You should report how often each experiment was performed
- You should include a definition of biological versus technical replication
- The data obtained should be provided and sufficient information should be provided to indicate the number of independent biological and/or technical replicates
- If you encountered any outliers, you should describe how these were handled
- Criteria for exclusion/inclusion of data should be clearly stated
- High-throughput sequence data should be uploaded before submission, with a private link for reviewers provided (these are available from both GEO and ArrayExpress)

Please outline where this information can be found within the submission (e.g., sections or figure legends), or explain why this information doesn’t apply to your submission:

In each experiment a sample size of at least N=2 was used, except one sample with N=1 described here: For ATAC-seq experiment, N=5 of tenocyte sample, N=4 of attachment cell sample and N=2 of chondrocyte sample were used. For bulk RNA sequencing, N=2 of 4 sample types were used, except adjacent chondrocytes transcriptome where N=1 was used and found to be similar to other samples in this study (i.e. transcriptome of remote chondrocytes, N=2). For scRNA-seq experiment N=2 was used. For *in situ* hybridization and single molecule fluorescent *in situ* hybridization experiments, N=2 were used. For transgenic mouse reporter enhancer assay experiments, N=2/9 for Col1a1 element (mm1995), N=3/3 for Klf2 element (mm1988), N=4/7 for Sox9 element (mm1989), N=3/3 for Mgp element (mm1990), N=11/11 for Col11a1 element (mm1991) were used, respectively. No technical repetitions are reported, therefore all samples reported are biological.

The high-throughput sequence data (Bulk RNA sequencing and ATAC-seq) was submitted to GEO (GSE144306), with the following private link for reviewers: <https://www.ncbi.nlm.nih.gov/geo/query/acc.cgi?acc=GSE144306>

token: ezudggcozpgrjcl.

The high-throughput sequence data (Single-cell RNA sequencing) was submitted to GEO (GSE160090), with the following private link for reviewers:

[https://www.ncbi.nlm.nih.gov/geo/query/acc.cgi?acc=GSE160090](https://www.ncbi.nlm.nih.gov/geo/query/acc.cgi?acc=GSE160090)

token: ijanmwmmntyrhuh

**Statistical reporting**

- Statistical analysis methods should be described and justified
- Raw data should be presented in figures whenever informative to do so (typically when N per group is less than 10)
- For each experiment, you should identify the statistical tests used, exact values of N, definitions of center, methods of multiple test correction, and dispersion and precision measures (e.g., mean, median, SD, SEM, confidence intervals; and, for the major substantive results, a measure of effect size (e.g., Pearson's r, Cohen's d)
- Report exact p-values wherever possible alongside the summary statistics and 95% confidence intervals. These should be reported for all key questions and not only when the p-value is less than 0.05.

Please outline where this information can be found within the submission (e.g., sections or figure legends), or explain why this information doesn’t apply to your submission:

Exact p-values and statistical tests are reported in the Results and Methods parts of the manuscript:

1. For ATAC sequencing analysis, p-values calculations are described on page 11, as part of the description of Fig.3A-C, and on page 17 as part of the description of KLF2/4 binding sites in ATAC-Seq peaks associated with the regulatory regions of 374 genes, that were shown to be expressed by attachment cells (Results).

2. For bulk RNA sequencing analysis, p-values calculations are described on page 34, under Bulk RNA sequencing (Methods).

3. Single-cell RNA sequencing analysis is described on page 35-36, under scRNA-seq bioinformatic analysis (Methods).

(For large datasets, or papers with a very large number of statistical tests, you may upload a single table file with tests, Ns, etc., with reference to sections in the manuscript.)

**Group allocation**

- Indicate how samples were allocated into experimental groups (in the case of clinical studies, please specify allocation to treatment method); if randomization was used, please also state if restricted randomization was applied
- Indicate if masking was used during group allocation, data collection and/or data analysis

Please outline where this information can be found within the submission (e.g., sections or figure legends), or explain why this information doesn’t apply to your submission:

Group allocation was not relevant to our study, which did not involve study groups.

**Additional data files (“source data”)**

- We encourage you to upload relevant additional data files, such as numerical data that are represented as a graph in a figure, or as a summary table
- Where provided, these should be in the most useful format, and they can be uploaded as “Source data” files linked to a main figure or table
- Include model definition files including the full list of parameters used
- Include code used for data analysis (e.g., R, MatLab)
- Avoid stating that data files are “available upon request”

Please indicate the figures or tables for which source data files have been provided:

Figure 1 and Figure 3; raw RNA-seq files in addition to ATAC-seq files were deposited in GEO (GSE144306). Pipeline and software that were used in this study are described at the Methods part.

Figure 2; high-throughput sequence data of Single-cell RNA sequencing was deposited in GEO (GSE160090). Pipeline and software that were used in this study are described at the Methods part.
